# A More Aesthetically Pleasing Smile: A Case Report on the Impact of Scalpel Technique on Gingival Depigmentation

**DOI:** 10.7759/cureus.66144

**Published:** 2024-08-04

**Authors:** Khushee Raisoni, Amit Reche, Motilal Jangid, Priyanka Paul, Anushka Jain, Komal Agrawal

**Affiliations:** 1 Periodontics, Sharad Pawar Dental College, Datta Meghe Institute of Higher Education and Research, Sawangi, Wardha, IND; 2 Public Health Dentistry, Sharad Pawar Dental College, Datta Meghe Institute of Higher Education and Research, Sawangi, Wardha, IND; 3 Periodontics, Saraswati Dhanwantari Dental College and Hospital, Parbhani, IND

**Keywords:** case report, patient satisfaction, aesthetically pleasing smile, scalpel technique, gingival depigmentation

## Abstract

Gingival hyperpigmentation can significantly affect how the smile looks cosmetically, leading patients to seek treatment. This case report addresses the use of a scalpel approach combined with bur abrasion for gingival depigmentation in a 19-year-old female patient who was displeased with her "black" gums. A local anesthetic was required for the surgical removal of the pigmented epithelium and a thin layer of connective tissue. Post-operative care included antibiotics and analgesics, with chlorhexidine mouthwash use for optimal healing. Follow-up examinations showed successful depigmentation without complications. This simple method is a good choice for gingival pigmentation management since it provides satisfactory cosmetic results and patient satisfaction.

## Introduction

Together with tooth position, color, and form, gingival tissues are essential to preserving the harmony of the smile. A beautiful grin must include both the health and beauty of the gingiva [1]. Nowadays, patients and the public at large are increasingly aware of aesthetics. Patients with gingival hyperpigmentation often complain about their black gums, which presents a cosmetic issue [2]. Melanin-induced pigmentation is a natural and physiological process [3,4]. Patients often complain of "black gums," especially if they have a very high smile line, despite the fact that melanin pigmentation is entirely benign and poses no health risks [5]. The prevalence rate of gingival depigmentation was 40% [6]. The term "aesthetic" indicates beauty rather than just pleasingness, meaning that the most desired qualities are present. Achieving optimal oral beauty involves. The gingival appearance is one of the many elements that affect colour; it can vary in shades from pale to dark brown or black. It is believed to be connected to cutaneous pigmentation and varies throughout individuals [7]. Distinctive features of gingival pigmentation include strands of dark purplish discolouration, irregularly formed patches, and diffuse brown, light brown, or black spots [3]. Melanin granules are the end product of melanoblastic production. Melanin, the most common natural pigment, is a dark pigment that is not derived from haemoglobin. Melanocytes produce melanin pigment, which are located in the basal and suprabasal cell layers of the epithelium. Gingival hyperpigmentation is the outcome of too much melanin being deposited in the basal and supra-basal cell layers of the epithelium.

In certain populations, hyperpigmentation is considered a hereditary variable that is unrelated to age or gender [8]. Clinically, oral mucosa melanin pigmentation can be diffuse or multifocal, exhibiting a range of clinical characteristics, including physiological alterations (like ethnic pigmentation) as well as systemic disease symptoms (like Addison's disease) and malignant tumours (like melanoma and Kaposi's sarcoma). Gingival depigmentation, a plastic surgery procedure in the realm of dentistry, involves using a variety of procedures to eliminate or lessen gingival pigmentation [9]. By means of a scalpel, a layer of the underlying connective tissue and the gingival epithelium is surgically extracted, allowing secondary intention healing to occur. Within the recently developed epithelium, melanin pigmentation is absent [10]. Numerous methods, including de-epithelization, electro-surgery, cryosurgery, chemical agents, and lasers, are available for depigmentation [11].

This case study presents a straightforward and efficient surgical depigmentation method that produces cosmetically pleasing outcomes and patient satisfaction without the need for complex equipment.

## Case presentation

Patient information

**Figure 1:**
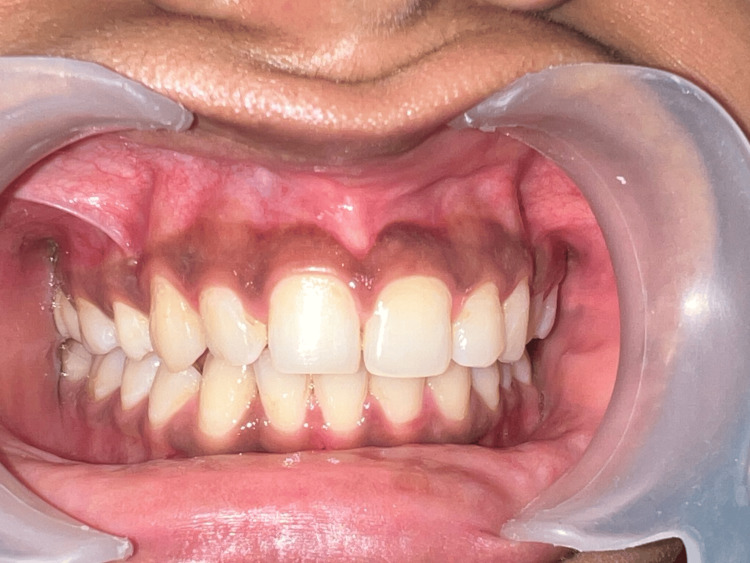
Preoperative picture of a 19-year-old woman who complained of having "black" gums

A 19-year-old female patient who appears to be in good systemic health arrived at the Department of Periodontology with a major complaint of "black" coloured gum as visible in Figure 1. Her gingiva was found to be significantly pigmented from the right to the left first premolar during her oral examination. The woman desired any aesthetic procedure that would improve the appearance of her "black" gums

**Figure 2:**
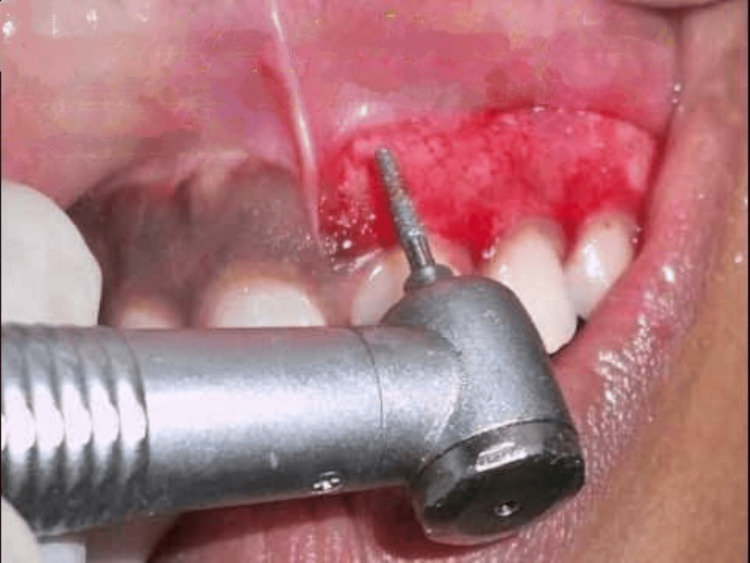
Bur abrasion on pigmented gingiva

**Figure 3:**
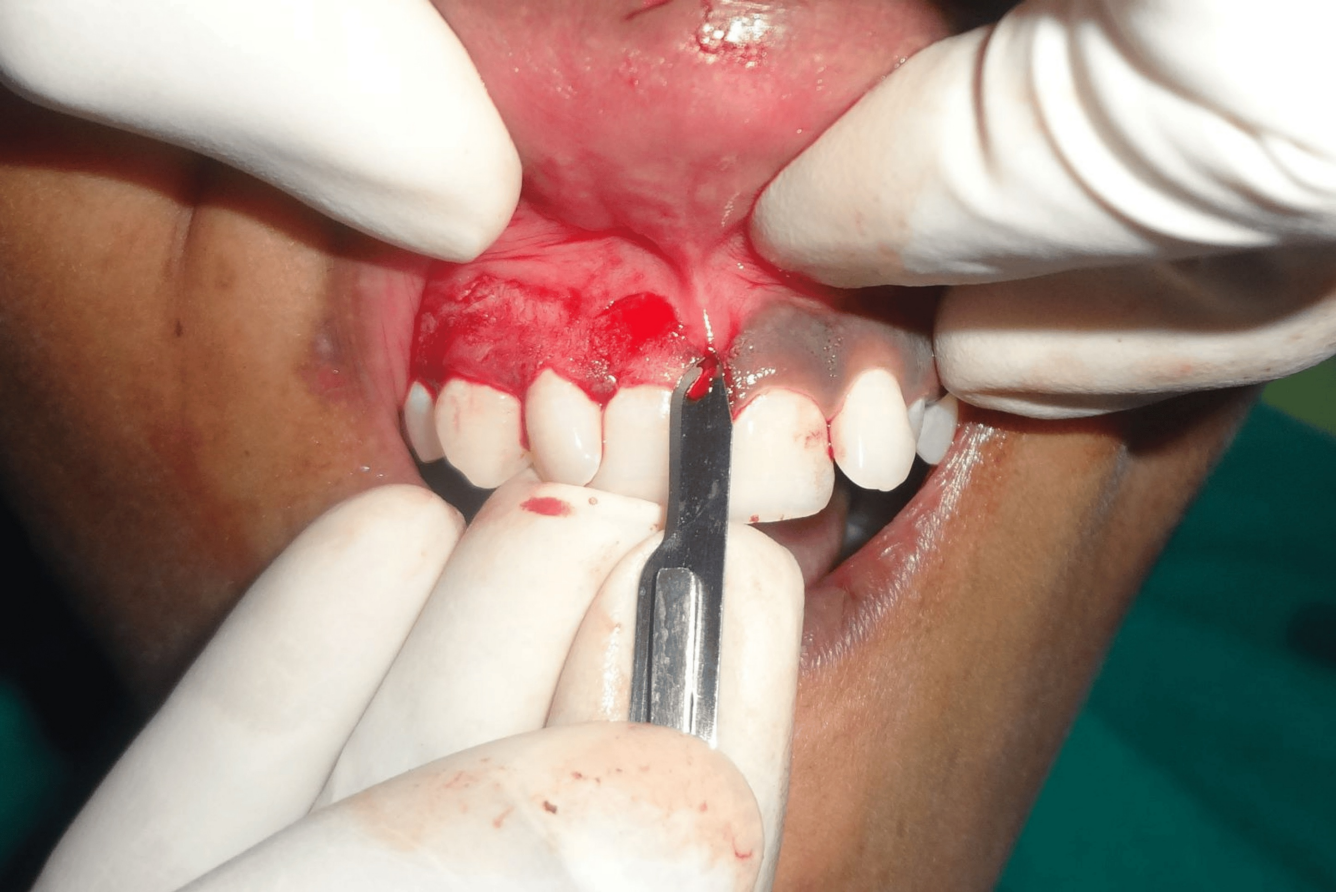
The maxillary anterior gingiva's pigment layer removed using the number 15 blade

To accomplish the depigmentation, a scalpel procedure with bur abrasion was scheduled. Written consent was acquired after the patient was fully informed about the surgery. Blood testing, family history, and a comprehensive medical assessment were done to rule out any surgical contraindications. From premolar to premolar, the maxillary anterior area was injected with local anesthetic agent (lignocaine with adrenaline in the ratio 1:100000 by weight). Using a high-speed handpiece with a diamond bur as shown in Figure 2 and a Bard Parker grip with a No. 15 blade as demonstrated in Figure 3, the highest pigmented layer of gingiva was removed while maintaining alignment with the teeth's long axis. The least amount of pressure and force was applied to prevent gingival pitting after surgery.

**Figure 4:**
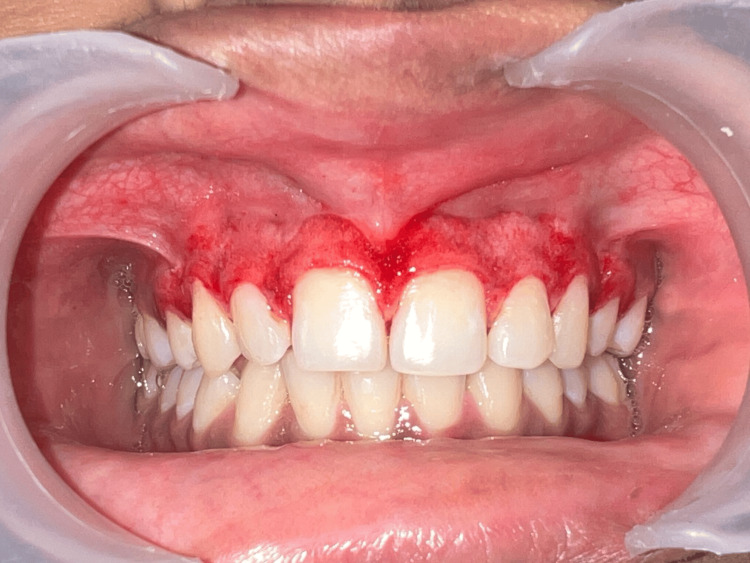
Removal of maxillary anterior gingiva's pigmented layer.

**Figure 5:**
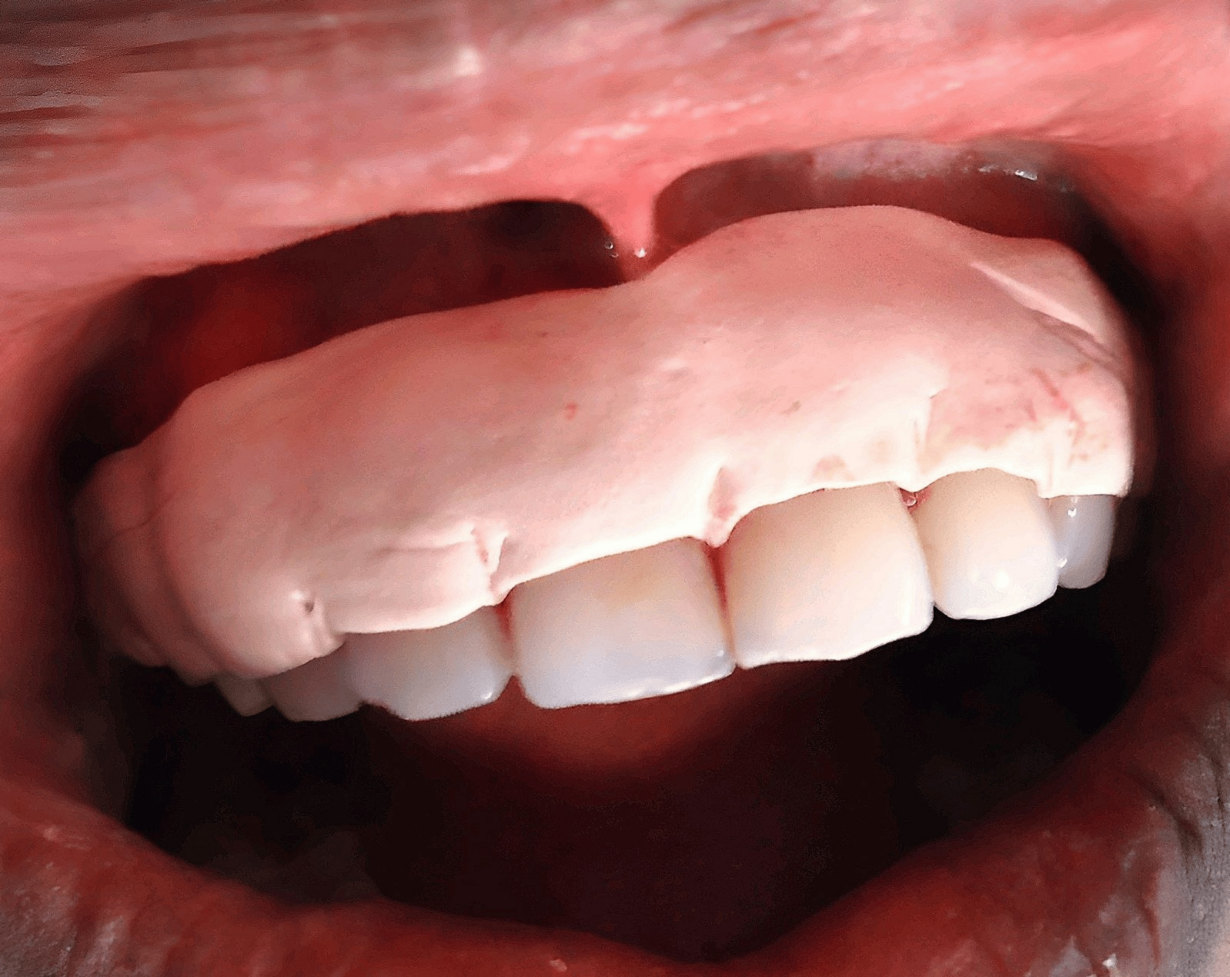
Periodontal dressing covering the operative area

Throughout the procedure, sterile gauze soaked in a local anesthetic was used to cease the bleeding and apply pressure. The gingiva was abrased with a diamond bur to obtain its physiological contour, and then the entire pigmented epithelium and a thin layer of connective tissue were removed using a scalpel as shown in Figure 4. Next, saline was used to irrigate the exposed region. The bur was used with the least amount of pressure possible, using feather-light brushstrokes and not keeping the bur in one place. Every effort was made to ensure that the pigmented layer's remnants were eliminated. Over the surgical site, a periodontal dressing was applied as displayed in Figure 5.

**Figure 6:**
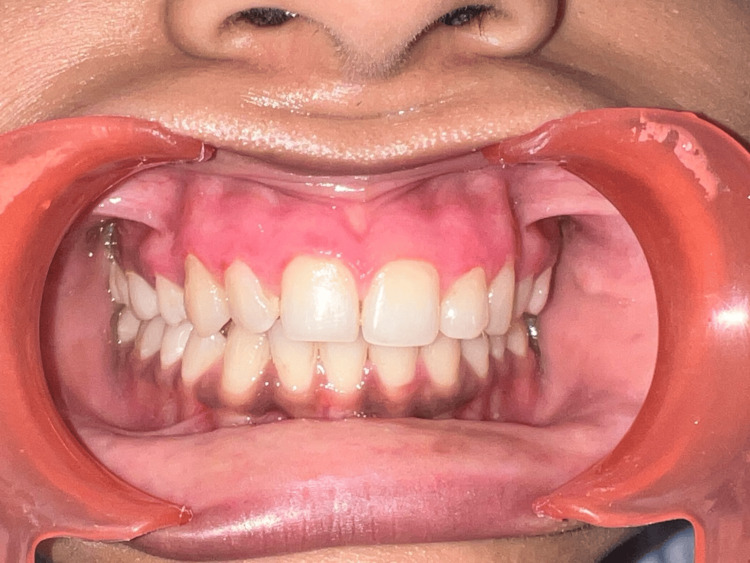
One week post-operative picture

After surgery, the patient was given antibiotics (amoxicillin 500mg, three times a day for five days) and analgesics (ibuprofen with paracetamol, three times a day for three days) along with vitamin E application. For a week, the patient was told to use mouthwash containing chlorhexidine every 12 hours. Following the initial week, the patient underwent review. Following the removal of the periodontal dressing pack, the surgical site was examined. The patient reported no pain, and the healing process was going according to plan. There were no post-surgical problems. Follow-up was taken after one week as elaborated in Figure 6.

The patient was directed to take the mouthwash containing chlorhexidine for an extra week. A month later, after re-epithelialization, the healing was satisfactory. After the procedure, the patient reported no pain or sensitivity. After 90 days (three months), the patient was summoned back to assess the progress of recovery. Gingival melanin pigmentation was observed for clinical recurrence and its intensity. In addition to being entirely free of irritation and recurring pigmentation, the gingiva had a solid consistency and looked pink and healthy.

## Discussion

The cosmetic look of the smile can be greatly affected by gingival hyperpigmentation, even if it is benign. This can prompt patients to seek treatment for what are commonly mistaken for "black gums."

This case study describes how gingival pigmentation was successfully managed with a scalpel approach and bur abrasion, leading to both patient satisfaction and an improvement in appearance. In the method outlined in this case study, under local anesthetic, a straightforward surgical procedure was performed to carefully remove the hyper-pigmented gingival epithelium and a thin layer of connective tissue. This procedure effectively achieves depigmentation by removing melanin-containing cells, therefore limiting recurrence and producing a more uniform pink appearance of the gingiva. The use of a scalpel and bur ensured precise removal of pigmented tissue, while minimizing trauma to surrounding tissues [12].

The most prevalent, dark endogenous pigment produced by melanocytes is melanin, which is found in the basal layer that is not derived from hemoglobin [13]. Each person's gingival melanin pigmentation differs in intensity, from a patchy to diffuse distribution, and depends on a range of parameters, particularly activity of melanoblast and densely packed melanophores (melanin-containing cells) in the gingiva.

In individuals with darker skin tones, the possibility of having pigmented gingiva is much higher than fair-skinned individuals [14]. Oral pigmentation in men and women does not differ substantially from one another. The amount and distribution of pigmentation in the oral mucosa differ not only across races but also within the same race and between different mouth regions. Though physiologic pigmentation might be genetically showing healthy gingiva with no areas of pigmentation determined, Dummett proposed that there is a correlation between the degree of pigmentation and physical, pharmacological, and mechanical stimulation [5]. The majority of melanocytes that are actively producing melanin pigmentation in the gingiva and oral mucosa as a whole are found in the oral epithelium's basal layer [15]. It is possible to eliminate pigmentations for cosmetic purposes. Various therapeutic approaches have been employed to achieve this goal [16]. When selecting a gingival depigmentation procedure, it is crucial to take the patient's financial situation, clinical experience, and personal preferences into account. More skill is needed for electro-surgery than for scalpel surgery. Applying electricity to tissue over an extended period of time or repeatedly causes heat buildup and undesirable tissue damage. Make sure not to come into contact with the periosteum, alveolar bone, or essential teeth [17]. Significant swelling and enhanced soft tissue damage are the negative effects of cryosurgery. Prolonged freezing accelerates tissue damage, even if it is impossible to control the depth and the ideal freezing time is unclear [8]. Laser depigmentation produces good results, but it is expensive, takes up a lot of space, and requires complex equipment. The pigmented regions can also be removed with a free gingival graft, but color matching and an additional surgical site (donor site) are needed [18]. However, these forms of treatment are not commonly recognized or employed. Given the limitations of the equipment, which are sometimes unavailable in clinics, the scalpel surgical approach is strongly advised [8]. Scalpel wounds are reported to heal more quickly than wounds treated with conventional methods. On the other hand, bleeding during and after scalpel surgery can be uncomfortable, and periodontal dressings must be used for seven to 10 days to cover the exposed lamina propria [8]. Gingiva post-surgery regimentation has been documented before.

Re-pigmentation is characterized as occurring on its own and has been linked to the migration and activity of surrounding melanocytic cells [18]. After one week in this instance, there was no sign of re-pigmentation. Three months later, no more re-pigmentation was seen. To measure the degree and pace of re-pigmentation even more, the case is being monitored.

## Conclusions

In addition to being an efficient surgical technique for gingival depigmentation, the scalpel approach in conjunction with bur abrasion yields good cosmetic outcomes and patient satisfaction. This process, which efficiently removes the melanin-containing cells and reduces recurrence, involves carefully excising the hyper-pigmented gingival epithelium and a thin layer of connective tissue while under local anesthesia. When it comes to managing gingival pigmentation, the scalpel method is a good alternative to more restrictive methods like electrosurgery, cryosurgery, and lasers since it ensures precise removal of the pigmented tissue with a minimal amount of damage to the surrounding tissues. The patient reported no pain or difficulties following the operation, and at least three months had passed since the effective depigmentation that was observed during periodic follow-up visits.
